# Thermodynamic Complexing of Monocyclopentadienylferrum (II) Intercalates with Double-Walled Carbon Nanotubes

**DOI:** 10.1186/s11671-016-1351-7

**Published:** 2016-03-08

**Authors:** О. V. Мykhailenko, Yu I. Prylutskyy, І. V. Кomarov, А. V. Strungar

**Affiliations:** Taras Shevchenko National University of Kyiv, Volodymyrska Str., 64, 01601 Kyiv, Ukraine

## Abstract

By employing the methods of molecular mechanics, semi-empirical quantum-chemical РМ3 and Monte-Carlo, the positioning of monocyclopentadienylferrum (II) molecules in double-walled (5,5)@(10,10) carbon nanotubes (CNT) depending on their concentration and temperature has been studied. The molecules have been found out to form stable bonds with CNT walls, with a tendency between intercalate stability and the CNT structure. The temperature growth (over ~500 K) causes gradual bond ruining followed by extrusion of interwall intercalate. Further temperature increase up to 600–700 K is characterised with intercalate external surface desorption, stabilising the whole system and keeping the interwall intercalate only. The CNT’s UV-spectrum (5,5)@(10,10) depending on the intercalate concentration and association constant of the “double-walled CNT–intercalate” system have been calculated. A combination of unique optical, electrical and magnetic behaviour of cyclopentadienyl complexes with their ability to form high-stable intercalate with CNT opens a prospect of their applying in nanotechnology.

## Background

Unique physical properties of multi-walled nanosystems (especially of graphene-based ones) have been the subject of keen interest lately. Their specific energy-band structure with a zero band gap and linear dependency of electron and hole energy spectrum from the wave-vector cause the electric charges to behave like relativist particles with zero effective mass [1–3]. Anomalous transportation and field effects open a wide prospect of their applying in nanoelectronics [4–7]. Such nanostructures are assumed to be promising spintronics materials due to the long electron-free path, weak spin-orbital interaction and the long spin scattering [8, 9]. What is more, the chemical or physical modification of multi-walled nanosystems enables to reveal their new extraordinary features. Thus, intercalation with atoms (molecules) allows to change the Fermi level position, relative electron and hole concentration, without considerable changes in energy-band structure of source nanomaterials [10–12].

On the other hand, unique optical, electrical and magnetic, and also biological behaviour of cyclopentadienyl complexes [13] stimulate creation on their base of intercalates with multi-walled carbon nanotubes (MWCNT), since the ability of these complexes to coordinate with MWCNT [6, 10] allows to obtain new materials (including catalytic methods with cyclopentadienyl catalysts [14]) as effective elements for photo- and magnetosensitive devices.

As identification of structure-to-property relations is an important task of chemistry and material physics, the aim of this work was to study the structure of intercalated monocyclopentadienylferrum (II) double-walled CNT (DWCNT) under heating by the methods of molecular mechanics ММ+, semi-empirical quantum-chemical РМ3 and Monte-Carlo, to calculate the UV-spectrum of a DWCNT depending on intercalate concentration as well as to determine the association constant of the “DWCNT-intercalate” system.

## Methods

The initial structure was a (5,5)@(10,10) DWCNT having 270 carbon atoms. Intercalation of this DWCNT assumes placing the intercalate inside the (5,5) CNT, into intertubular space, and its differently oriented sorption on the outer surface of a (10,10) CNT.

**Fig. 1:**
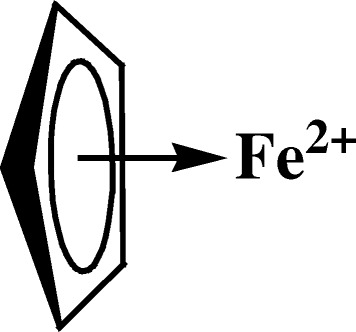
Monocyclopentadienylferrum (II) molecule as the intercalate

The intercalate is the monocyclopentadienylferrum (II) molecule (Fig. 1).

It is their relative position, orientation relatively the CNT walls, behaviour in the intertubular space and the system as a whole, as well as the quantity characteristics of bonding with the DWCNT at temperature change that is the subject of this calculation.

In the model considered the interaction potential between Fe^2+^ ions (see Eq. (1)) and a cyclopentadienyl anion directly mated the pair potential of high energy of atomic excitation [15], and it was described by the Born-Mayer equation within 0–0.2815 nm of effective interaction radius (see Eq. (2)).

1 $$ U(r)=4\varepsilon \left[{\left(\frac{\sigma }{r}\right)}^{12}-{\left(\frac{\sigma }{r}\right)}^6\right], $$

where *r* is the distance between particle centres, *ε* is the depth of potential pit and *σ* is the distance at which the interaction energy is equal to zero (parameters *ε* and *σ* characterise atoms of corresponding substances);

2 $$ U<<{E}_0\approx \frac{\hslash^2}{m{r}^2}, $$

where *m* is the mass of a particle.

To describe the atom interaction at a distance smaller than 0.2 nm, there was a use of the Tersoff-Brenner potential of interatomic interaction [16]. Total potential energy of system *U* is expressed as a sum of bonding energies for all pairs of atoms forming this system.

3 $$ U={\displaystyle \sum_i{\displaystyle \sum_{j>i}\left[{V}_R\left({r}_{ij}\right)-{B}_{ij}^{*}{V}_R\left({r}_{ij}\right)\right]}}, $$

where *r*_*ij*_ is the distance between *i* and *j* atoms; *V*_*A*_(*r*) and *V*_*R*_(*r*) are the exponential functions of the Morse potential type which corresponds to the energies of attraction and repulsion between the atoms; and *B*_ij_^*^ is the function expressing the dependence of binding energy of the *і* and *j* atoms from the angles *θ*_*ijk*_ between the bond *i-j* and close bonds *i-k* and *j-k*.

To describe the atom interaction at a distance greater than 0.21 nm, the Tersoff-Brenner potential of interatomic interaction was employed [16] along with the Ziegler-Biersack-Litmark pair potential [15]. The length of С–С bonds in a CNT was 0.139 nm, the Fe–C interaction was described by the Lennard-Jones pair potential [17] with potential interaction energy 0.12 eV. The modelled period of one excitation cascade was 2 ps, and the energy conservation law in every calculation cycle was correlated within 0.15 %. The initial coordinates of the intercalate were selected in conformity with the law of random numbers.

To do the task above, the following numerical modelling scheme was used [10, 11]:

1. The first calculation stage was based on the ММ+ molecular mechanics method;
2. The second stage was based on the semi-empirical РМ3 method. It should be noted that the main distinction of this method from the others is their different parameterisation. In our case, РМ3 method was parameterised to the best match of calculated and experimental molecule formation heats;
3. The third stage was based on the Monte-Carlo method.

To calculate the association constant of the “DWCNT-intercalate” complex formed, the modified Benes-Hilderbrand method [18] that accounts the data on the maximum DWCNT absorption values at various intercalate concentrations in the UV-spectrum was employed.

## Results and Discussion

Modelling the “DWCNT–intercalate” system resulted in such statements. First, the four intercalate molecules in the intertubular space form a high-temperature resistant system—up to ~475 K, being completely and quickly extruded at higher temperatures. Second, two of the four monocyclopentadienylferrum (II) molecules placed beside the outer side surface of the (10,10) CNT are desorbed at the temperature ~600 K. The other two intercalate molecules are within the effective interaction radius, remaining sorbed up to the temperature ~750 K. Third, all the monocyclopentadienylferrum (II) molecules being in the inner (5,5) CNT are stable regardless of the temperature factor. Last, the two sorbed intercalate molecules which were not desorbed from the outer side surface of the (10,10) CNT were oriented at the latter one by the cyclopentadienyl ring, but not by Fe^2+^.

**Fig. 2:**
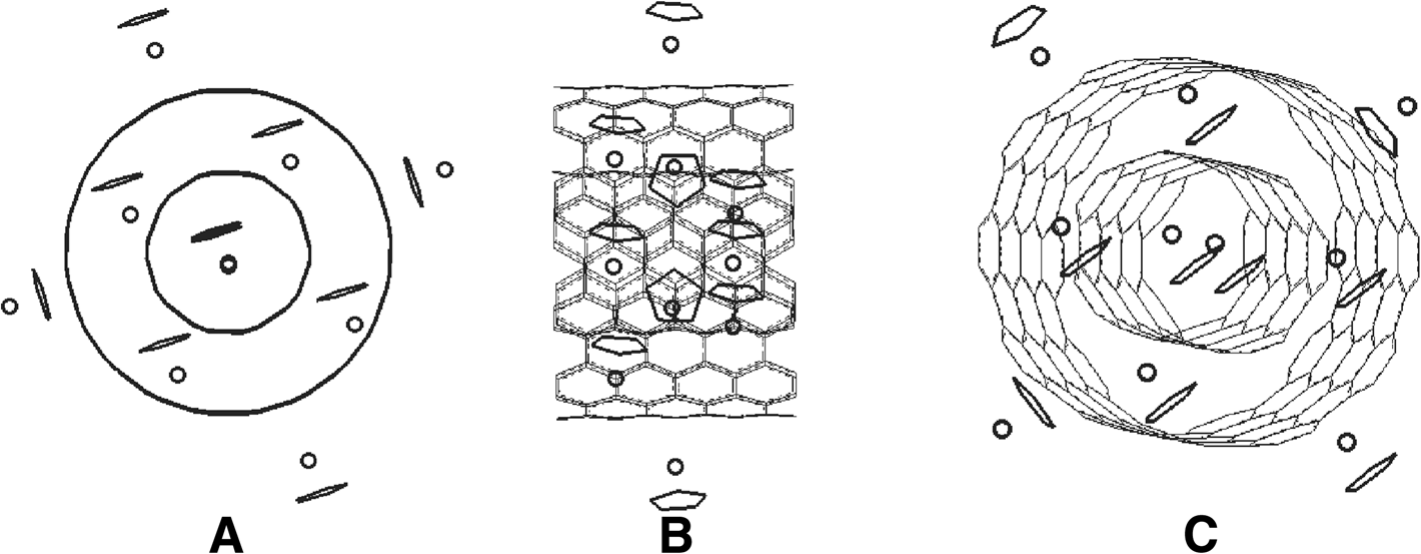
Geometric model of the “DWCNT-intercalate” system: **a**, **b** are the orthogonal projections; **c** is a side-view

The studied system (see Fig. 2) proved to be rather heat-resistant in a wide temperature range (up to ~900 K). At that, the deformation vibrations of the DWCNT’s crystal grate do not exceed 0.015 nm, and the vibrations of the intercalate molecules do not exceed 0.025 nm that provides for configuration and conformation stability of the system.

**Fig. 3:**
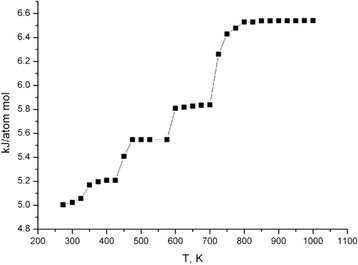
Temperature dependence of the “DWCNT-intercalate” model system

Temperature dependence of the model system energy is shown in Fig. 3. As it is seen, when initially heated from 273 to ~425 K, the system energy grows gradually and then rises sharply between 425–475 K and 725–750 K, then, with temperature growth, it reaches the plateau that proves its high stability up to ~900 K.

**Fig. 4:**
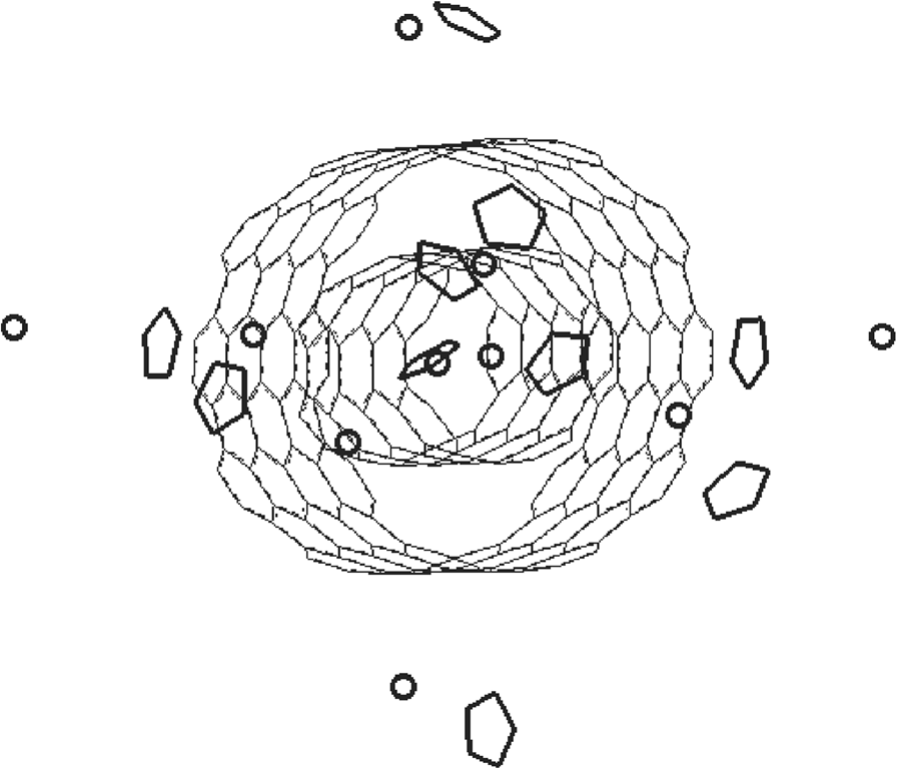
Screenshot of configurational change of the “DWCNT-intercalate” system under heating

The modelling performed allows to determine the following dynamics of the intercalated DWCNT structure under heating: when being initially heated to ~425 K, the system remains rather stable; there is no extrusion of the intercalate molecules. There are observed vibrational and rotational (along the quintic axis) capabilities of bonds and angles of a DWCNT and monocyclopentadienylferrum (II) molecules. When the system temperature is increased to ~500 K, the rapid intercalate extrusion out of the intertube space of the system appears. At the temperature of ~600 K, the desorption from the outer surface of only two intercalate molecules being ferrum-oriented to the DWCNT wall is observed. At the temperature of over ~800 K, the full external desorption appears while the inner (5,5) CNT remains completely filled (Fig. 4 shows the screenshot of configurational change of the “DWCNT-intercalate” system under heating).

It should be noted that this variant of the model suggested allows to observe thermodynamic selectivity of physical and chemical sorption-desorption. At the temperature range 273–425 K, the physical sorption whose natural feature is most likely to overlap the non-hybrisized orbital 3*d*_*xy*_ of the Fe^2+^ ion with the π-system of the DWCNT side surface appears while chemisorption is observed at higher temperature values (~600 K), that is peculiar or π-π interactions of aromatic and quasiaromatic cyclic and heterocyclic systems.

Moreover, simultaneous presence of donor/acceptor feature of the DWCNT’s intertube space as a result of positive and negative Gaussian curvature makes it possible to regulate orientation of intercalate’s donor and acceptor edges, which allows to view it as a potential molecular switch.

**Fig. 5:**
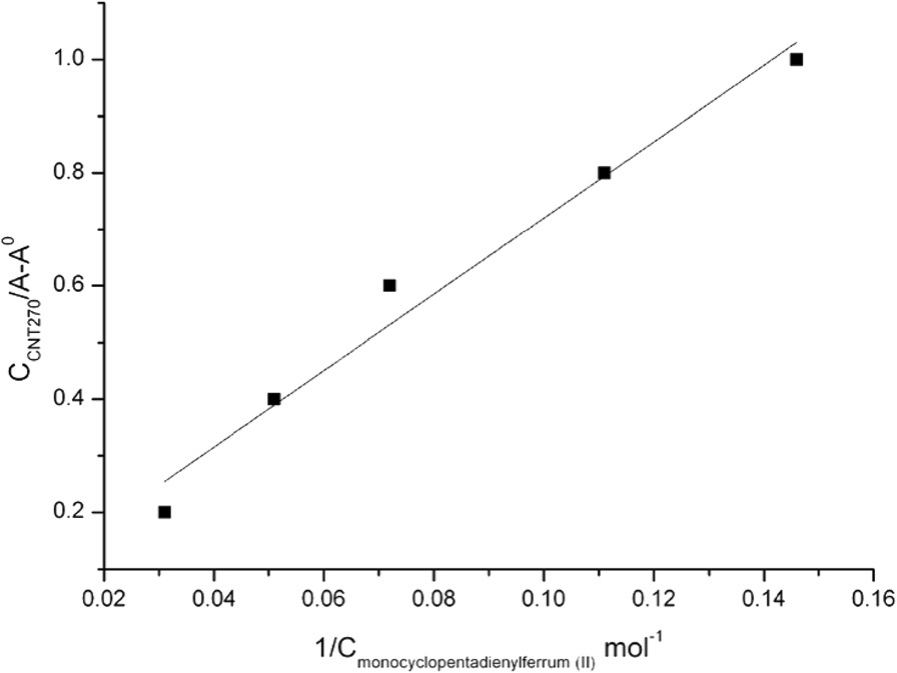
Dependence of DWCNT’s absorption with intercalate added in Benes-Hilderbrand coordinates

Finally, theoretical calculations of UV-absorption spectra of the (5,5)@(10,10) DWCNT depending on the intercalate concentration in terms of the modified Benes-Hilderbrand method (Fig. 5) show that the association constant of the system studied is 6.745 l · mol^−1^.

## Conclusions

The type of monocyclopentadienylferrum (II) molecule arrangement on the outer side surface and on the inner surface as well as in the intertube space of the (5,5)@(10,10) DWCNT has been found. The calculations made allow to prove discover that the “DWCNT-intercalate” system is rather stable at high temperature (up to ~425 K) that provides reliability and stability of the process of intercalate synthesis under conditions regular for this procedure. However, at further heating (over ~500 K), the gradual intercalate extrusion out of the intertube space and intercalate’s outer surface desorption (at the temperature 600–700 К) as well as thermodynamic stabilisation of the system containing the intertube intercalate only are observed. The association constant of the “DWCNT-intercalate” system has been theoretically calculated, and it is equal to 6.745 l · mol^−1^.
